# Inferring pandemic growth rates from sequence data

**DOI:** 10.1098/rsif.2011.0850

**Published:** 2012-02-15

**Authors:** Eric de Silva, Neil M. Ferguson, Christophe Fraser

**Affiliations:** Department of Infectious Disease Epidemiology, MRC Centre for Outbreak Analysis and Modelling, Imperial College London, London W2 1PG, UK

**Keywords:** phylodynamics, epidemics, simulation

## Abstract

Using sequence data to infer population dynamics is playing an increasing role in the analysis of outbreaks. The most common methods in use, based on coalescent inference, have been widely used but not extensively tested against simulated epidemics. Here, we use simulated data to test the ability of both parametric and non-parametric methods for inference of effective population size (coded in the popular BEAST package) to reconstruct epidemic dynamics. We consider a range of simulations centred on scenarios considered plausible for pandemic influenza, but our conclusions are generic for any exponentially growing epidemic. We highlight systematic biases in non-parametric effective population size estimation. The most prominent such bias leads to the false inference of slowing of epidemic spread in the recent past even when the real epidemic is growing exponentially. We suggest some sampling strategies that could reduce (but not eliminate) some of the biases. Parametric methods can correct for these biases if the infected population size is large. We also explore how some poor sampling strategies (e.g. that over-represent epidemiologically linked clusters of cases) could dramatically exacerbate bias in an uncontrolled manner. Finally, we present a simple diagnostic indicator, based on coalescent density and which can easily be applied to reconstructed phylogenies, that identifies time-periods for which effective population size estimates are less likely to be biased. We illustrate this with an application to the 2009 H1N1 pandemic.

## 1. Introduction

With the growth of faster and more reliable sequencing technologies (and consequently the availability of genetic data), there have been a number of statistical and computational innovations to analyse this proliferation of sequence data. One successful application has been the analysis of epidemic trends using the so-called phylodynamic methods that use pathogen sequences to infer pathogen diversity, and the changing number of infected individuals as well as more subtle effects on pathogen selection and population structure [1,2]. For a recent review of methods used in such analyses, see Ho & Shapiro [3] and references therein.

These methods typically use coalescent approaches [4]. The coalescent is a framework that predicts distributional aspects of branch lengths in temporal phylogenetic trees in terms of a demographic parameter called the effective population size. A temporal tree is one where emergence times for internal nodes are estimated (assuming tips have known isolation dates). If the mutation rate is known (or can be estimated), then a temporal scale can be superposed on to the phylogenetic tree. As one goes back in time through the tree, lineages coalesce at points called coalescent events and there comes a time when all the lineages coalesce into a single lineage: this is the most recent common ancestor and the time to it from the present is known as the time to the most recent common ancestor (TMRCA). If the evolving organisms are not under strong selection during the time up to their TMRCA and there is no population structure, then the effective population size is expected to be approximately equal to the actual population size (in practice, it is almost always less than the actual population size).

More specifically, coalescent analysis starts from the straightforward observation that for the Wright–Fisher model, the probability that two randomly selected individuals from one generation share a parent is equal to 1/*N*, where *N* is the population size. Taking the definition of the effective population size, *N*_e_, to be the size of a Wright–Fisher model population that would give an observed level of diversity, we can then observe for an arbitrary panmictic population, the probability that two lineages in one generation have a common ancestor lineage in the previous generation is equal to 1/*N*_e_. Applying this observation to a phylogenetic tree, which encodes the ancestral probability distribution of all coalescent event times in the population, provides estimates of the changing effective population size throughout the history of the population up to the TMRCA.

The classical skyline plot [5] (see also the electronic supplementary material) allows a non-parametric estimation of the past effective population size to be made. The generalized skyline plot [6] reduces the noise and stochasticity found in classical skyline plots by grouping coalescent events over which changes in effective population size are estimated. Each interval will therefore have a number of coalescent events as well as sampling events (points in time at which sequences are collected).

Bayesian evolutionary analysis by sampling trees (BEAST) is a popular software package that integrates many phylogenetic and coalescent-based tools [7]. It can be used to estimate phylogenetic trees and apply the coalescent model to infer demographic changes from sequence data. A Bayesian approach is used: given the data (aligned sequences), a specified model structure (coalescent, substitution model, rate heterogeneity model) and specified prior distributions of parameters, the posterior distribution of trees and parameters is estimated. BEAST uses an efficient Markov chain Monte Carlo (MCMC) algorithm to sample the posterior distribution of phylogenetic trees and parameters. As a result of this approach, the time-varying effective population size estimated by the coalescent is averaged over many phylogenetic trees. If a non-parametric estimate based on the generalized skyline plot is implemented, then the resulting estimate is called the Bayesian skyline plot (BSP). Both piecewise constant and piecewise linear skyline models can be implemented, the latter appropriate if one knows *a priori* that the inferred population is growing. It is also possible to specify specific parametric models for the effective population size, such as exponential growth, which may be appropriate when analysing an emerging epidemic. We will explore both approaches here.

During the recent pandemic of H1N1 influenza in 2009, such techniques were used fruitfully in the early phase of the epidemic to estimate the rate of spread and the likely date of first emergence of this virus in the human population [8,9]. In common with all RNA viruses, H1N1 influenza has a high mutation rate and short generation time such that genetic changes encode epidemiological information. The combination of these properties allow use of both sequence data and temporal information obtained early on in the pandemic to compute infection dynamics while the pandemic is still progressing.

**Figure 1.**
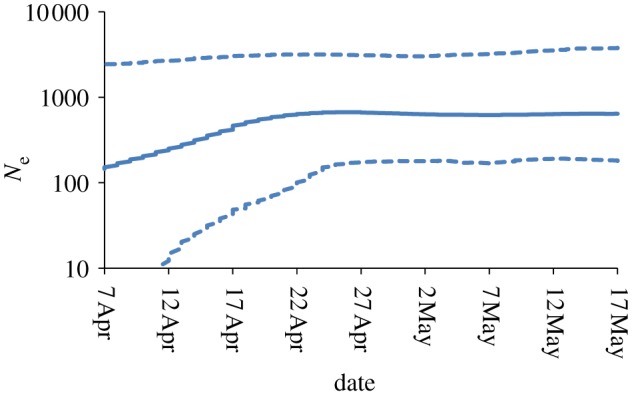
Bayesian skyline plot (BSP) for H1N1 (2009) for United States sequences collected between 20 April 2009 and 24 May 2009 (downloaded from GenBank NCBI on 10 September 2010). Owing to large variation in the numbers of viral samples sequenced over time, we sampled 22 sequences each week over this five week period (no attempt was made to account for possible clusters), giving 110 in total (for accession numbers, see the electronic supplementary material). Solid line shows median *N*_e_ estimates; dashed lines show the 95% credible intervals (CI). The BSP shows an increase in *N*_e_ throughout April followed by a slowdown towards the end of April. The TMRCA is 85 (CI: 48–161) days (i.e. 28 February 2009; CI: 6 April 2009–14 December 2008) and the substitution rate is estimated to be 1.97 × 10^-5^ (CI: 7.78 × 10^−6^ to 3.23 × 10^−5^) sub per site per day.

As an example, figure 1 shows a retrospective BSP generated using BEAST (for temporal frequency, see the electronic supplementary material, figure S1) of the effective population size of 2009 H1N1 derived from the 110 publically available complete haemagglutinin (HA) viral sequences (excluding duplicates) listed in the NCBI influenza virus resource [10] as arising from specimens collected in the USA over the period 7 April 2009 to 24 May 2009. This analysis covers a seven week period at the start of the pandemic, and thus extends the initial analyses presented in Fraser *et al*. [8]. Figure 1 shows a slowdown in the growth rate of the effective population size around late April/early May. If this reflects slowing growth in the number of infected individuals, then this would be of epidemiological interest—perhaps suggesting some impact of public health measures enacted in the USA at that time.

Indeed, this flattening in the latter portion of the growth curve of the effective population size is visible in BSPs looking at HIV [11–13], dengue [14] and hepatitis C [15], and is often interpreted as being evidence for a slowing of spread. However, the implication that H1N1 transmission may have temporarily been reduced around late April 2009 should be treated with caution. Samples obtained in the USA during spring 2009 will be beset with sampling biases: spatially (more samples from some areas than others, given that US states varied in the level of testing undertaken), temporally (more intensive testing was often undertaken for the first few weeks of cases in a locality, and less thereafter, and there were national changes in testing protocols) and epidemiologically linked (samples taken from individuals in the same local outbreak, and which therefore are not random independent samples from the H1N1 infected population overall). In some analyses, a few of these biases were controlled for by removing sequences collected from patients in known local epidemiological clusters [8] but in general it is not clear the extent to which such biases in sampling affect estimates.

To further understand the impact of these biases, and more generally to validate methods for imputing epidemic growth rates from sequence data, we simulated epidemics broadly similar to the H1N1 pandemic, subjected simulated datasets to many of the same biases as real data, and applied coalescent-based approaches to estimate the effective population size over time.

Recently, Stack *et al*. [16] have performed a similar exercise for seasonally fluctuating influenza epidemics, and showed how different sampling protocols can influence inferred transmission dynamics in BSPs. In the case of seasonal epidemics, they point out the importance of temporal sampling, especially where populations undergo bottlenecks and the number of lineages are substantially reduced from one season to the next. BSPs inferred from samples taken after a bottleneck are unable to recover transmission dynamics prior to the bottleneck. Their analysis indicates that single-generation sampling or sampling randomly about a target generation as the epidemic begins to slow down is the most effective strategy. Also of interest is their observation that joining together successive BSPs inferred over shorter time-scales was more representative of changes in population size than a single BSP over a longer time period, which they suggest may be owing to poorly specified prior probabilities in BEAST. Certainly for longer-term population dynamics strongly influenced by selection from the immune system or vaccination, the use of coalescent methods becomes questionable.

For the work presented here, we adopted a branching process model to simulate the early stages of an epidemic. The model is parametrized by the basic reproduction number, *R*, which determines the rate of (exponential) growth of the epidemic, a dispersion parameter, *k,* which quantifies heterogeneity in infectiousness between individuals, and the generation time. We incorporate a realistic mutational model so that starting from a sequence of one of the early isolates, we simulate sequences collected over a specified number of generations of spread in the epidemic. We then compare these to assess how well coalescent methods are able to reliably estimate the true dynamics of the simulated epidemic.

## 2. Material and methods

### 2.1. Simulation model

The model is seeded with a starting sequence of arbitrary length—in this case, the full-length HA gene of one of the first US H1N1 influenza virus isolates A/California/04/09. The seed sequence is generation 0 at time *t* = 0 and its offspring comprise generation 1 and time, *t* = *T*_g_ and their offspring generation 2 and time *t* = 2*T*_g_ and so on, where *T*_g_ is the generation time. Rambaut *et al*. [17] find the HA gene to have mean rate of evolutionary change to be 5.72e^−3^ nucleotide substitutions (sub) per site per year (the highest of all the proteins, unsurprisingly, given its function in avoiding the immune system). This equals 1.57e^−5^ sub per site per day, which for a generation time of 2.6 days is 4.1e^−5^ sub per site per generation. In our simulation, we choose a mutation rate of 1.83e^−4^ sub per site per generation, faster than the estimate of Rambaut *et al.*, as the substitution rate for many pathogens is expected to be faster at short time scales owing to the long-term effects of purifying selection [18]. Our findings were not very sensitive to the mutation rate (analysis not shown).

Generations of the simulated epidemic are discrete. In each generation, the number of offspring generated by each sequence of the current generation is evaluated by sampling a negative binomial distribution with mean *R* (which can potentially change over time) and dispersion parameter *k* (which is held fixed over time). Each nucleotide of each sequence of the next generation is either inherited directly from its ancestor or mutates from its parent with some probability. If a nucleotide does mutate, which type of substitution occurs is drawn from a substitution matrix derived from the Kimura two-parameter model.

To keep our simulation simple, we did not consider epidemics evolving in continuous time. However, we did find that our findings were robust to halving the generation time and taking the square root of the reproduction number (analysis not shown), and as a result we hypothesise that simulations carried out in real-time would lead to very similar results.

We discuss the assumptions made in this algorithm below.

### 2.2. The offspring distribution

Following on from Lloyd-Smith *et al*. [19] and Grassly & Fraser [20], we introduce the individual reproductive number, *ν*, which is the expected number of secondary cases caused by a specific infected individual. If *ν* is gamma-distributed having a mean *R* and dispersion parameter *k*, then the number of secondary infections caused by each infected individual is Poisson distributed with mean *ν*, and thus overall the number of secondary infections generated by a single infection is given by a negative binomial offspring distribution. The probability *p*(*m*) of obtaining *m* from a negative binomial distribution with parameters *p* and *k* is given by:
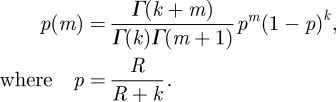


In general, lower values of *k* correspond to more heterogeneity in infectiousness, and thus epidemics are increasingly characterized by superspreaders. For *k* = 1, the distribution is geometric, while it becomes Poisson as *k* → ∞. We assume that the number of offspring each sequence generates is independent of and distributed identically to the offspring generated by any other sequence. Implicitly this is equivalent to assuming random (although not homogeneous) mixing and an effectively infinite susceptible population. For many parameter combinations (especially low *k* and/or low *R*), there is a high probability of early extinction of a simulated outbreak. Because we are interested in emerging epidemics, not self-limited outbreaks, we select our simulated datasets from outbreaks which do not go extinct.

### 2.3. The substitution model

While the branching process model assumes a fixed generation time, *T*_g_ of 2.6 days [21], in calculating nucleotide substitutions, we use a more realistic generation time distribution with gamma form [20,22]:
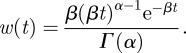
Here, *α* is the shape parameter, *β* the inverse scale parameter. We assume a mean generation time of 2.6 days and standard deviation 1.3 days [21], giving *α* = 4 and *β* = 1.

For simplicity, we assume a Kimura two-parameter substitution model [23] with transition mutation rate parameter *a* = 0.4 and transversion mutation rate parameter *b* = 0.2 [24]. The nucleotide transition probability matrix over time interval, *t* is:
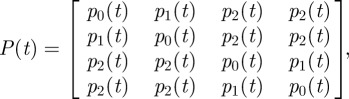
where
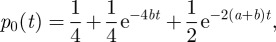

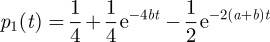

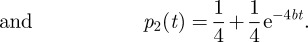


We wish to average this transition matrix over the generation time distribution. The resulting matrix has the Kimura form with elements defined by:
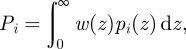
namely
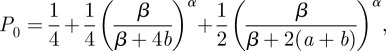

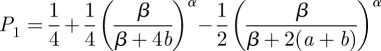

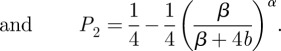


### 2.4. Sampling strategies

In order to assess how inferred BSPs are affected by the way in which viral sequences are collected from infected individuals, we examine two sampling schemes: uniform and log-proportional. Under uniform sampling, we randomly pick one sample per generation of simulated sequences and use these selected sequences to infer posteriors and BSPs. This would be analogous to choosing to sequence a virus from a randomly selected infected person at regular temporal intervals, perhaps set by laboratory capacity.

Under log-proportional sampling, we randomly extract samples in proportion to the logarithm of the number of samples available per generation. This is closer to how viral samples are collected during an epidemic where more samples are collected if more individuals are infected, but sampling proportional to the actual number of cases is not possible for logistical reasons.

We simulate viral spread under a reasonable range of values for the *R* and *k* parameters. We examine *R* = 1.5 (a typical estimate for the 2009 pandemic [8]) and in addition the higher values of 2.0 and 2.5 which may be more realistic for ‘typical’ previous influenza pandemics [25]. Because the dispersion parameter *k* is unknown, we consider a wide range, from extreme superspreading (such as thought to apply to the severe acute respiratory syndrome epidemic [19]) (*k* = 0.1) to more homogenous infectiousness (*k* = 1 and 10).

A feature of virological surveillance during an epidemic is that some geographically localized outbreaks can be heavily sampled, whereas others are sampled much less, if at all. This results in groups of sequences that are closely related (genetically) and thus no longer constitute a random sample of all sequences. In order to assess the effects of such non-random sampling—or samples that are epidemiologically linked (e.g. from the same family where family members have infected one another)—we sample along the branches of a single lineage. In this way, we sample the first offspring of the first offspring of the first offspring generation after generation all the way along the tree.

### 2.5. Bayesian evolutionary analysis by sampling tree input and output

Real and simulated HA sequences were first aligned using ClustalX [26] and the multiple alignments visually confirmed. The aligned sequences were then imported into BEAST together with the dates each sample was collected. Given the small temporal extent over which samples were collected, we used a nucleotide substitution model of Hasegawa *et al*. [27] (which subsumes the two-parameter Kimura model used to generate simulated data) to avoid over-parametrization. Mutation rate heterogeneity among aligned sites was described by a gamma distribution.

BEAST then uses the sequence alignment to construct a Jukes–Cantor distance matrix from which a starting unweighted pair-group method with arithmetic mean tree is made. A relaxed clock molecular model [28,29] where branch rates are drawn from an underlying lognormal distribution is assumed. For two parameter combinations, we verified that BEAST converged to the same equilibrium distribution of parameters for a range of randomly starting trees (analysis not shown).

The tuning parameters that control the speed and efficiency with which the MCMC chain used to sample tree space in BEAST equilibriates are automatically optimized. Required MCMC chain lengths are determined via evaluation of the effective sample sizes (ESSs) for the parameters of interest, and depend strongly on the number of sampled sequences. The ESS is the number of effectively independent draws from a marginal posterior distribution of a parameter that a particular length of MCMC chain is equivalent to. A low ESS for a given parameter implies high correlation between MCMC samples and that the posterior distribution will be poor. The software package ‘Tracer’ (http://tree.bio.ed.ac.uk/software/tracer/) is used to check ESSs and examine the trace for any trends that may indicate lack of convergence, or any long-range fluctuations indicative of poor mixing.

Each BEAST run is repeated at least twice so as to independently confirm convergence. The package Tracer is also used to plot the BSP and lineages-through-time (LTT) plots. The package ‘FigTree’ (http://tree.bio.ed.ac.uk/software/figtree/) is used to plot the maximum clade credibility (MCC) tree, defined as the tree with the highest sum of posterior probabilities of the internal nodes.

## 3. Results

The simulation generates full-length HA sequences for each infected individual. We simulate the first 100 000 infections of an epidemic, which for most simulations corresponds to 40 or more days in chronological time. For comparison, the empirical BSP in figure 1 covers the 40 days between 7 April and 17 May 2009.

### 3.1. Sampling density

**Figure 2.**
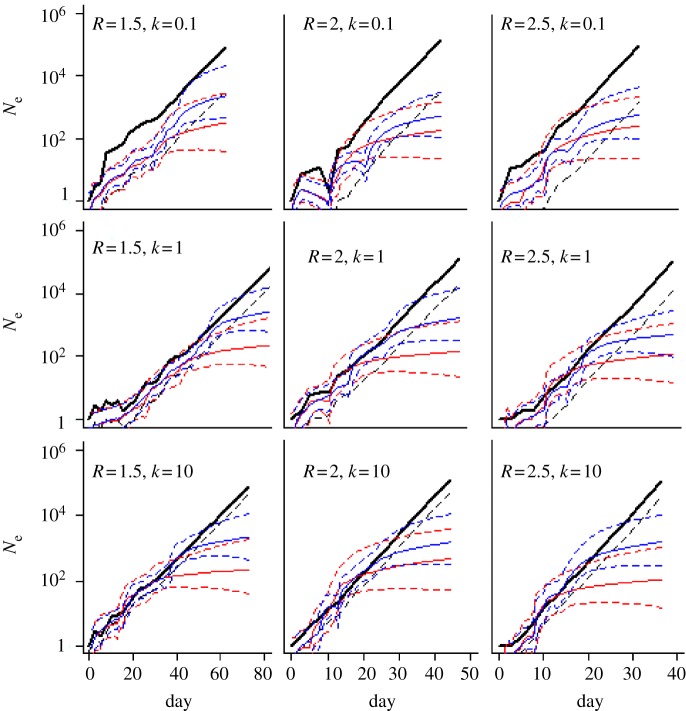
Bayesian skyline plots (BSPs) produced by sampling simulated data sparsely. Red curves show median *N*_e_ estimates when just one sequence is randomly sampled per generation, and blue curves show the corresponding estimates for a more dense sampling (number of sequences sampled per generation proportional to the log of the number of sequences in that generation—see text for details). Dashed lines show 95% credible intervals (CI). Results for nine parameter combinations for the underlying branching process model used to generate the data are shown (*R* = 1.5, 2, 2.5 and *k* = 0.1, 1, 10). Black lines show the true number of infected individuals per generation from the simulated data. Black dashed lines are the instantaneous effective population size found by correcting the simulated number of individuals infected, *N*.

The black curve in figure 2 shows the number of infected individuals, and thus the number of sequences generated, under a range of reproduction numbers and dispersion parameters. In all the plots, stochastic variation is visible during the early part of the epidemic. As expected, the growth rates become progressively higher for higher *R*-values. Additionally, the number of extinction events (runs in which the simulated population dies out in the first few generations owing to no more offspring) is greatest for the low *R*, low *k* parameter combinations (*R* = 1.5, *k* = 0.1). Table 1 summarizes details of these simulations and table 2 results from the Bayesian analysis.

**Table 1.** Details of simulations used to simulate the epidemics in figure 2.

*R*	*k*	total generations	simulation length (day)	sequences in final generation
1.5	0.1	24	62.4	81 591
1.5	1	33	85.8	97 258
1.5	10	28	72.8	74 607
2	0.1	16	41.6	121 802
2	1	18	46.8	119 088
2	10	17	44.2	109 924
2.5	0.1	12	31.2	87 917
2.5	1	15	39	102 563
2.5	10	14	36.4	105 127

**Table 2.** Details of sampling simulated epidemics and properties inferred from non-parametric Bayesian coalescent used to produce BSPs in figure 2.

sampling scheme	*R*	*k*	number of sampled sequences	TMRCA (day)	final *N*_e_
uniform	1.5	0.1	25	62.7 (62.4, 63.2)	323 (40, 2851)
proportional	1.5	0.1	81	62.6 (62.4, 63.1)	2374 (457, 20 936)
uniform	1.5	1	34	86.5 (85.8, 87.4)	232 (40, 1805)
proportional	1.5	1	94	86.6 (85.8, 87.4)	2813 (553, 17 503)
uniform	1.5	10	29	73.4 (72.8, 74.6)	213 (39, 1829)
proportional	1.5	10	85	73.4 (72.8, 74.6)	2129 (425, 11 071)
uniform	2	0.1	17	41.8 (41.6, 42.2)	177 (22, 1441)
proportional	2	0.1	52	41.7 (41.6, 42.2)	508 (102, 2883)
uniform	2	1	19	47 (46.8, 47.5)	144 (22, 1315)
proportional	2	1	56	47 (46.8, 47.4)	1658 (324, 14 059)
uniform	2	10	18	44.6 (44.2, 45.4)	444 (49, 3632)
proportional	2	10	55	44.5 (44.2, 45.4)	1403 (292, 10 095)
uniform	2.5	0.1	13	31.7 (31.2, 32.5)	250 (22, 2074)
proportional	2.5	0.1	41	31.8 (31.2, 32.7)	562 (97, 4158)
uniform	2.5	1	16	39.1 (39, 39.4)	117 (15, 1094)
proportional	2.5	1	45	39.1 (39, 39.5)	494 (97, 2827)
uniform	2.5	10	15	36.5 (36.4, 36.9)	104 (14, 985)
proportional	2.5	10	43	36.6 (36.4, 36.9)	1464 (270, 9593)

The black dashed line in figure 2 is the effective population size estimated directly from the simulated number of infected individuals, using the known relationship between census population size, effective population size and variance of offspring distribution [4,30,31]. Using the formula for the negative binomial distribution given earlier, the ratio of effective population size (*N*_e_) to census population size (*N*) is equivalent to the reciprocal of the variance (*σ*^2^), which can be written in terms of the reproduction rate (*R*) and offspring distribution (*k*):
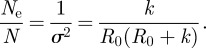
Figure 2 indicates that estimates of the effective population size obtained using BEAST tend to be biased upwards in the early phase of the epidemic, before they tend to level off.

The red curves in figure 2 are BSPs generated via uniform sampling. While these estimates effectively capture epidemic dynamics over the earlier generations, by the halfway point of the epidemic, growth in the BSP curves begin to slow down significantly. The final effective population size is thus significantly less (this discrepancy being more pronounced for higher *R*-values) than the actual size of the simulated infected population, leading to a false inference of a slowing epidemic.

Similarly, the blue curves graph the BSPs generated from the same simulated sequences via log-proportional sampling. These estimates were therefore generated with many more sequences (typically around three times more; table 2). The *N*_e_ estimates obtained under this sampling scheme capture the exponential growth of the epidemic better than those obtained from uniform sampling, but there is still a slowdown in estimated growth rates during the latter stages of the epidemic. The gains in accuracy achieved by denser sampling are modest, and the bias of apparent flattening towards the present, visible in all simulations, is only modestly reduced.

### 3.2. Coalescent events

**Figure 3.**
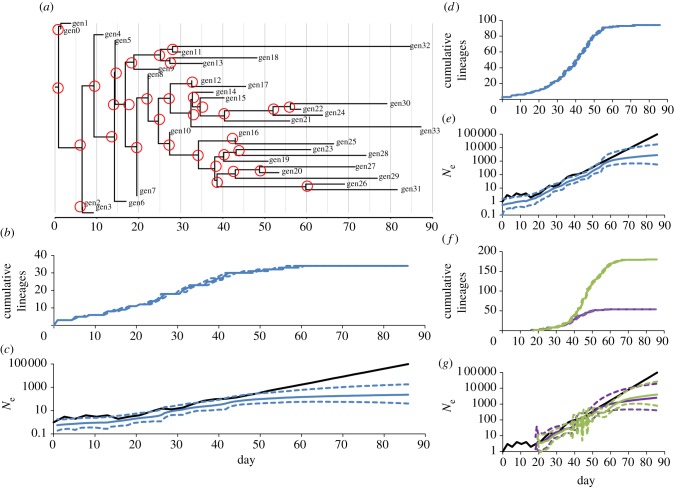
(*a*) Maximum clade credibility (MCC) phylogenetic tree obtained from a Bayesian coalescent analysis of 34 randomly sampled sequences (one per generation of the simulated epidemic), generated from a simulated population with *R* = 1.5 and *k* = 1. Coalescent events are encircled in red (including the final coalescent event) and tip labels give the epidemic generation from which the sequence was drawn. (*b*) Cumulative lineages through time (LTT) plot of same dataset with credibility intervals (CIs). (*c*) Corresponding Bayesian skyline plot (BSP; blue lines) with 95% confidence intervals, and black line showing true number of infections over time. (*d*) Cumulative LTT plot for the log-proportionally sampled *R* = 1.5 and *k* = 1 simulated dataset in figure 2 and (*e*) corresponding BSP. (*f*) Cumulative LTT plot for regular and denser sampling of three (purple lines) and 10 (green lines) sequences per generation; sampling random and from generations which have more than 100 sequences (figure 5*b*). (*g*) Corresponding BSP (black line represents simulated sequences).

Given that the estimated changes in effective population size are inferred from the reconstructed phylogeny, it is worth taking a look at the corresponding phylogenetic tree. Consider figure 3*c* which shows the BSP inferred from 34 randomly sampled sequences (one per generation) taken from a simulated viral population with *R* = 1.5 and *k* = 1 using a piecewise-linear skyline model. The black line shows the true number of infections (and simulated sequences) through time. As shown in figure 2, the BSP gives growth rates that slow at later times. Figure 3*a* shows the corresponding MCC tree similarly scaled (with time increasing to the right) to the BSP. The red circles highlight the coalescent events in this tree. Going backwards in time a coalescent event marks the merging of lineages and going forwards in time it represents the creation of new lineages. The generalized skyline plot—of which the BSP is an example—groups coalescent events into time intervals which are bound by either a coalescent event or a sampling event and within which changes in effective population size are estimated. These changes are a function of the number of lineages present and the MCC tree clearly shows that the final (most recent) coalescent event coincides with the flattening of the BSP. This is clearly illustrated by looking at the LTT plot, which counts the cumulative number of LTT (figure 3*b*). This flattens where there are no longer any new lineages (i.e. at the time of the last coalescent event), coincident with the flattening of the BSP. The lack of genealogical information at later times is also visible in the increased size of the highest posterior density confidence intervals in the BSP over this period.

This then provides an explanation for why the BSPs flatten at later times: for an exponentially growing viral population (as is the case early on in an epidemic and in the simulated datasets we have been exploring) the phylogenetic tree is ‘star-like’. That is to say, it has long-terminal branches as in the tree in figure 3. This means that the final coalescent event will occur quite some time before the final sampling event (in figure 3*a*, there are 11 sampling events after the last coalescent event: gen23–gen33). From this period onwards, there are no new lineages (see LTT) and therefore no change in the effective population size is imputed. This issue (a form of left censoring in the ‘backwards in time’ coalescent context) represents a limitation to how far forwards in real-time the BSP can confidently be used to estimate *N*_e_. In light of this, we suggest that the BSP could be truncated at the time of the last coalescent event, rather than at the time of the last sample collection event.

The sharpness of the transition in the flattening of the LTT curve in figure 3*b* is striking and figure 3*d* illustrates the effect of sampling density on the shape of the LTT. Figure 3*d*,*e* are the LTT and BSP, respectively, of the log-proportionally sampled *R* = 1.5, *k* = 1 simulated dataset in figure 2 using a piecewise-linear skyline model. The greater sampling density still results in a relatively sharp transition in the LTT, and the BSP starts to flatten at the same time as the LTT slope flattens and the period after which there are no new lineages corresponding to little growth on the BSP. Figure 3*f*,*g* shows the LTT and BSP that result from randomly sampling respectively three and 10 sequences per generation (for generations with more than 100 sequences—see later). As expected, increasing the sampling density means the cumulative LTT slows down at later times corresponding to the last coalescent event and the time at which the BSP also slows down and/or flattens.

The Bayesian skyline assumes that *N*_e_ is autocorrelated through time and so smooths the vector representing the effective population size in each grouped interval [32]. We also experimented with varying the number of intervals (exploring group sizes of 5, 10, 20 and 30) but found very little difference in the BSPs produced (results not shown). While here we fixed the number of grouped intervals *a priori*, other methods use the reversible jump MCMC sampling [33].

Finally, we comment on the issue of why the 95% credibility intervals (CIs) do not overlap the actual or effective samples sizes towards the end of the simulation. Strictly, the skyline plot is an estimate of the harmonic mean of the effective population size over an inter-coalescent interval [5]. When the inter-coalescent periods are large, as they are in the end of the simulation, CIs on the estimate of the harmonic mean of the effective population size need not overlap with the actual value over the whole interval, and non-overlap does not represent a technical problem with the estimation.

### 3.3. Non-random sampling

**Figure 4.**
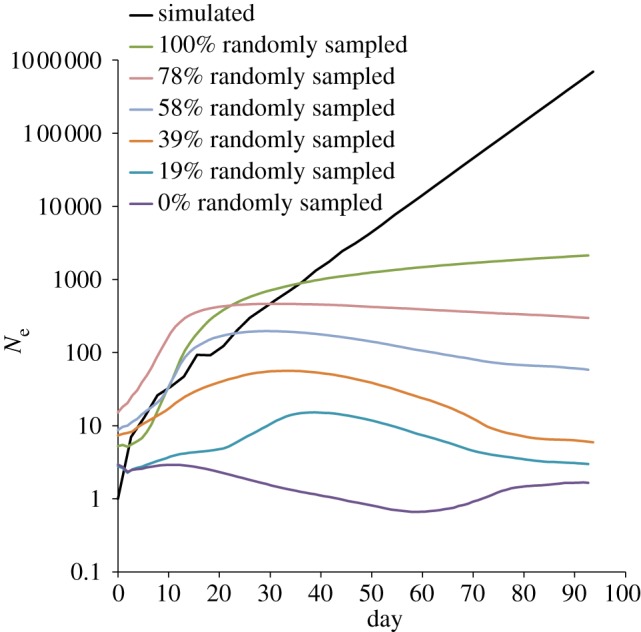
Simulated epidemic over 36 generations with *R* = 1.35 and *k* = 1. Black shows true numbers of infections (sequences) over time, coloured lines show Bayesian skyline plots (BSPs; median estimates of *N*_e_) obtained by sampling randomly (and uniformly) for a range of random and non-random (epidemiologically linked) sampling densities. Non-random (0% randomly sampled) sampling is effectively flat and close to one.

Figure 4 shows the effects on the BSP of sampling increasingly more epidemiologically linked sequences from a simulated epidemic. Here, we simulate an epidemic with *R* = 1.35 and *k* = 1 over 36 generations, with the resulting epidemic incidence curve shown in black. The purple curve (0% randomly sampled) shows the BSP inferred by sampling (one sequence per generation) from just one lineage, that is one direct descendent generation-to-generation. These closely related samples then represent an epidemiologically linked cluster and the resulting BSP is very poor at capturing the changes in the effective population size. In comparison, the green curve (100% randomly sampled) is the BSP inferred by sampling the same simulated population but this time completely randomly (once again one sequence per generation). The intermediate curves represent intermediate degrees of randomly sampled epidemiologically linked sequences. The final effective population size is proportional to the percentage of random sampling and only in the 100 per cent randomly sampled BSP is there continued growth (although small) beyond the last coalescent event (for corresponding LTT plot, see the electronic supplementary material).

### 3.4. Parametric versus non-parametric estimates of the growth rate

Because the problem we have described is that of censoring, and because we are analysing exponentially growing epidemics, we tested whether a parametric (exponential) model improves estimates. We generated a large number of simulated epidemics from three sets of branching process parameters (*R* = 1.15, *k* = 10 for *r* = 0.05; *R* = 1.35, *k* = 1 for *r* = 0.13; *R* = 1.7, *k* = 1 for *r* = 0.2), and then selected one representative simulation for each set of parameters which gave a realized epidemic growth rate *r* (measured using least-squares fit to log incidence) which matched that expected analytically in the infinite time (i.e. deterministic) limit. This selection step aimed to limit the impact of demographic stochasticity on incidence curves at the start of the simulated epidemic. The three resulting datasets had real-time growth rates of *r* ≈ 0.05, *r* ≈ 0.13 and *r* ≈ 0.2, respectively. For each of these simulated datasets, sequences were randomly sampled per generation log-proportionally, and the BSPs were estimated from these sequences. This random sampling was then repeated four more times (on the same original simulated epidemics) so that five sampled sequence sets were generated for each simulation (table 3; we are not interested here in exploring between-simulation variation).

**Table 3.** Simulated and corresponding Bayesian coalescent non-parametric and exponential inferred growth rates for log proportional sampling per generation and log-proportional sampling of generations with more than 100 sequences per generation (delayed sampling).

simulated growth rate	non-parametric Bayesian MCMC estimated growth rate	exponential coalescent estimated growth rate	delayed sampling: non-parametric Bayesian MCMC estimated growth rate	delayed sampling: exponential coalescent estimated growth rate
0.053	0.056	0.056 (0.048, 0.064)	0.051	0.054 (0.042, 0.065)
0.053	0.05	0.055 (0.046, 0.063)	0.046	0.048 (0.037, 0.059)
0.053	0.049	0.048 (0.04, 0.057)	0.046	0.046 (0.036, 0.059)
0.053	0.054	0.053 (0.044, 0.062)	0.052	0.052 (0.041, 0.064)
0.053	0.057	0.053 (0.048, 0.061)	0.052	0.05 (0.039, 0.061)
0.127	0.137	0.124 (0.106, 0.143)	0.13	0.118 (0.095, 0.141)
0.127	0.137	0.13 (0.112, 0.149)	0.127	0.121 (0.097, 0.145)
0.127	0.152	0.139 (0.119, 0.159)	0.137	0.126 (0.102, 0.15)
0.127	0.157	0.137 (0.117, 0.156)	0.132	0.126 (0.101, 0.15)
0.127	0.135	0.123 (0.105, 0.141)	0.128	0.117 (0.094, 0.139)
0.225	0.255	0.243 (0.194, 0.294)	0.228	0.22 (0.169, 0.277)
0.225	0.257	0.236 (0.186, 0.289)	0.225	0.209 (0.157, 0.262)
0.225	0.234	0.216 (0.169, 0.264)	0.204	0.226(0.149, 0.251)
0.225	0.273	0.23 (0.186, 0.281)	0.232	0.214 (0.162, 0.266)
0.225	0.269	0.268 (0.214, 0.324)	0.224	0.21 (0.154, 0.261)

We analysed these datasets with BEAST using the same non-parametric model for the growth of *N*_e_ as earlier, and then estimated the growth rate by fitting an exponential curve to the portion of the BSP that showed near exponential growth (truncating the curve following the last coalescent event). The resulting non-parametric estimates of the growth rates along with CIs for the parametric estimates are shown in table 3. The same samples were also analysed using BEAST to estimate an exponential growth rate for a parametric coalescent model with assumed exponentially growing effective population size.

**Figure 5.**
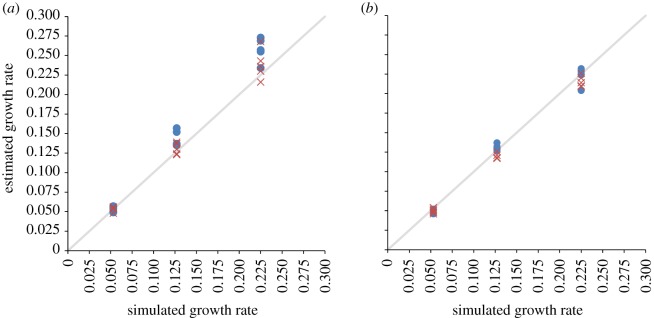
(*a*) Parametric and non-parametric (blue circles) Bayesian Markov chain Monte Carlo (MCMC) estimates of growth rate from simulated exponentially (cross symbols) growing epidemics with real-time growth rates of *r* ≈ 0.05, *r* ≈ 0.13 and *r* ≈ 0.2. Estimates repeated five times for each rate, each time randomly and proportionally sampling per generation of the epidemic. For non-parametric estimates, the region over which growth rate is estimated from the BSP is restricted to the region for which near exponential growth is seen. CIs not shown for clarity (table 3). (*b*) Same methodology as previous but this time randomly sampling proportionally from generations that have more than 100 sequences.

Figure 5*a* and table 3 show the resulting estimates of growth rates against the true values for the growth rate for each of the sampled sets. Even after correcting for the artificial flattening of the BSP, the exponential parametric model appears to be more accurate at estimating growth rates. The larger spread in estimated growth rates for higher simulated growth rate is probably a result of the larger truncation of BSPs owing to earlier flattening of LTT corresponding plots, and the corresponding loss of information on coalescence in the phylogeny.

Significantly, repeating all of the above, but this time only sampling sequences (still randomly and log-proportionally) from generations in which there are more than 100 sequences (figure 5*b*) results in much improved parametric and non-parametric estimates of growth rate than when sampling all generations. This is because a key assumption of the coalescent is broken when the number of infected individuals is small. Kingman's formulation of the coalescent assumes a maximum of one coalescent event in the sample per generation, meaning that the sample has to be a small proportion of the total population. During the early generations, a small number of infected individuals (or sequences in the population) are simulated. Here, the population is small, and therefore the sampling density high, explaining the poorer estimates in figure 5*a*.

### 3.5. Application to real H1N1 data

**Figure 6.**
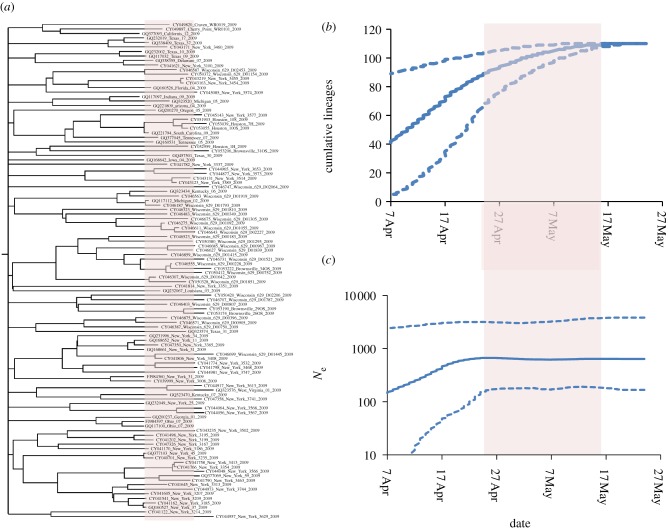
United States HA H1N1 data from April and May 2009 as detailed in figure 1. (*a*) The maximum clade credibility (MCC) tree for the 110 sequences sampled. (*b*) Lineages-through-time (LTT) plot from BEAST. (*c*) Bayesian skyline plot (BSP) plot, identical to figure 1 but shown here for comparison. The pink region on all plots shows the period between the end of growth in *N*_e_ estimated from the BSP and the point at which no new lineages are added to the LTT.

Now that we have some understanding of why the BSPs produced from sampling simulated data show a slowdown in effective population size for exponentially growing epidemics, we return to the case of the real H1N1 epidemic. The MCC phylogenetic tree and LTT plot can be examined to see whether the slowdown in the effective population size seen in figure 1 is likely to be an artefact of inference method (as seen in our analysis of simulated data), or instead reflects a real slowing of the epidemic. The dates on the LTT and BSP shown in figure 6 are aligned and the pink-shaded region represents the time over which the BSP estimates of *N*_e_ stops increasing to the point where there are no new lineages in the LTT. There are many coalescent events over this period in the MCC tree in agreement with the cumulative LTT, which increases over this period. This, therefore, validates the changes seen in the BSP in figure 1, and suggests that there may well have been a slowing in epidemic spread in late April and early May 2009, during the period when public concern about the new virus was most pronounced. Of course, other biases in the sampling that could confound the analysis, as described earlier, cannot be ruled out.

## 4. Discussion

While sampling density and the sampling of epidemiologically linked sequences both bias estimates of changes in effective population size, use of the Bayesian coalescent methodology results in a more notable bias in non-parametric estimates of changes in population size associated with the fact that for an exponentially growing genealogy, the density of coalescent events thins some considerable time before the latest sequence collection date.

Our results offer some support for the hypothesis that the inferred slowing of growth in the effective population size of H1N1 seen in the USA during late April/early May 2009 revealed by the BSP shown in figure 1 is not an artefact. However, given the very real impact sampling epidemiologically linked sequences has on inferred growth rates (as highlighted in our non-random sampling of simulated sequences), increasing epidemiological linkage in samples being collected cannot be ruled out as a contributory cause of the inferred slowing of epidemic growth. Future use of these methods would therefore be considerably aided by more detailed contextual epidemiological data on individuals from whom virological samples are collected and sequenced.

As with any Bayesian analysis where there are parameters having little or no prior information, there exists the possibility that the posterior estimate is being unintentionally biased by the chosen priors. However, we have explored the use of different priors (as well as other MCMC proposals within BEAST) and find that all the main results reported here are robust to such sensitivity analysis. We also note that the recently developed Bayesian ‘skyride’ [29] method places less emphasis on priors using Gaussian Markov random fields to smooth the effective population size over time.

The branching process model that we used to simulate epidemic data is deliberately simple, and lacks a number of important evolutionary and epidemiological features—notably population structure and selection. However, early in a pandemic when there is exponential growth of incidence (i.e. when numbers of infected are a very small proportion of total population size), we would not expect selection to play a major role. In addition, molecular analysis of the substitutions seen in the H1N1 virus in the first weeks of the epidemic give little support to the hypothesis that there were significant phenotypic changes that may have affected fitness. This is our major justification for assuming neutrality in the work presented here––but it should be noted that such an assumption would be expected to have more dubious validity later in a pandemic, when immunity, vaccine and antiviral use influence viral evolution. Of course, the presence of selection would also limit the applicability of coalescent methods to infer population dynamics.

Population structure would also not be expected to have a major impact very early in an epidemic, even in earliest affected communities, incidence by the end of May would not have been expected to have grown to the extent that epidemic growth rates would have slowed in some geographical areas but not others. Microstructure (e.g. households and local contact networks) is crudely represented in our model by allowing for highly over-dispersed offspring distributions.

Strictly, the ordinate axis of BSPs produced by BEAST is a compound value of *N*_e_. *τ*, where *τ* is the average generation time. In reality, the generation time varies from one infection to the next and so it is not straightforward to precisely evaluate *N*_e_ at a given time (some refer to this compound value as the genetic diversity); this is not the case for the simulations in this study where the generation time was fixed to *τ* = 2.6 days (and we use this value to show *N*_e_ in all plots). The interpretation of the absolute value of the estimated effective population size, *N*_e_, and what relationship this quantity has with the actual number of individuals infected is not obvious. While the effective population size is significantly less than the number of individuals infected towards the end of the simulation (this is not the case early on), this number increases with sampling density. In most studies, *N*_e_ is assumed to be the ideal (Wright–Fisher) population size that gives the same coalescent rate seen in the imputed phylogeny. That *N*_e_ is invariably less than the true population size is often attributed to population structure or other heterogeneity, such as in infectiousness. In addition, in this study, we quote the median posterior *N*_e_ estimates, which tend to be significantly smaller than the mean. Recent studies [34] conclude that the coalescence rate is a function of the prevalence, through sampling effects, as well as the incidence, although for a short generation time disease such as influenza, the difference between these is limited (effectively just a factor of the mean generation time). This study has focused more on the relative rate of change of *N*_e_ estimates than the meaning of the absolute values of such estimates, however.

Owing to the way in which BSPs are computed, a slowing or even flattening off at later times will often be present and can easily be misinterpreted as evidence for a slowing of epidemic spread. While this may not be a problem for computing the demographic history of many populations, it will be most significant when applied in real-time to an epidemic when the infected host population is increasing exponentially. If one knows *a priori* that the number infected is growing exponentially, then coalescent estimation can be improved by a parametric exponential model (as for analysing H1N1 influenza in Fraser *et al*. [8]) or even by using a more detailed epidemic model [35]. Another interesting approach that has recently been proposed is to replace Kingman's coalescent by a birth–death model, which perhaps better describes the process of infectious disease transmission [36]. This method would probably have the same advantage in accuracy as the exponential growth model, and may also avoid the problems observed when the infectious population is small (or more precisely when the proportion sampled is not small). This method should be included in future simulations.

When using the more flexible and non-parametric BSP approach, the times of coalescent events in the corresponding phylogenetic tree need to be examined (especially the time of the most recent coalescent event). Alternatively, the LTT graph should be inspected to estimate the time after which there are no new lineages. We suggest that any changes evident in the BSP from this time period onwards should be disregarded. This applies most strongly during the exponential growth phase of disease spread when the imprint of such growth will be reflected in long-branch lengths at later time periods in the phylogeny.
